# Usability Assessment of an Innovative Device in Infusion Therapy: A Mix-Method Approach Study

**DOI:** 10.3390/ijerph17228335

**Published:** 2020-11-11

**Authors:** Pedro Parreira, Liliana B. Sousa, Inês A. Marques, Paulo Santos-Costa, Sara Cortez, Filipa Carneiro, Arménio Cruz, Anabela Salgueiro-Oliveira

**Affiliations:** 1 Health Sciences Research Unit: Nursing (UICISA:E), Nursing School of Coimbra (ESEnfC), 3046-851 Coimbra, Portugal; parreira@esenfc.pt (P.P.); paulocosta@esenfc.pt (P.S.-C.); acruz@esenfc.pt (A.C.); anabela@esenfc.pt (A.S.-O.); 2 Biophysics Institute, Faculty of Medicine, University of Coimbra, 3000-354 Coimbra, Portugal; ines.marques@student.uc.pt; 3 Coimbra Institute for Clinical and Biomedical Research (iCBR), Area of Environment Genetics and Oncobiology (CIMAGO), Faculty of Medicine, University of Coimbra, 3000-354 Coimbra, Portugal; 4 CNC.IBILI (Center for Neuroscience and Cell Biology and Institute for Biomedical Imaging and Life Sciences), University of Coimbra, 3000-354 Coimbra, Portugal; 5 Muroplás-Plastic Engineering Industry, 4745-334 Muro, Portugal; sara.cortez@muroplas.pt; 6 PIEP—Innovation in Polymer Engineering, 4800-058 Braga, Portugal; f.carneiro@piep.pt

**Keywords:** usability, medical devices, double-chamber syringe

## Abstract

*Background*: Flushing a venous access device is an important procedure to maintain their patency and prevent malfunctioning and complications. An innovative double-chamber syringe was developed, allowing for the assessment of catheter patency, drug delivery and final flush. This study aims to assess the usability of this new device, considering three development stages (concept, semi-functional prototype, functional prototype). *Methods:* An iterative methodology based on a mix-method design (qualitative and quantitative) enabled the assessment of the devices’ usability by their primary end-users. A usability questionnaire was developed and applied, along with focus groups and individual interviews to nurses. *Results:* The usability questionnaire integrated 42 items focused on four dimensions (usefulness; ease of use; ease of learning; satisfaction and intention to use). The initial psychometric findings indicate a good internal consistency and the conceptual relevance of the items. The scores seem to be sensitive to the usability evaluation of the medical devices in different stages of product development (with lower values on functional prototype evaluation), and related to nurses’ perceptions about functional and ergonomic characteristics. *Conclusions*: Quantitative and qualitative data provided a comprehensive overview of the double-chamber syringes’ usability from the nurses’ point of view, informing us of features that must be addressed.

## 1. Introduction

Peripheral intravenous catheters (PIVC) are the most used invasive devices in hospitals [1,2], with over a billion PIVC being inserted each year in hospitalized patients worldwide [3]. However, there is a wide range of complications associated with PIVC insertion [4,5,6], such as phlebitis [7,8,9], accidental removal, occlusion [10] and bloodstream infection [11,12,13]. Several PIVC complications can lead to their malfunctioning, followed by their premature removal and performance of subsequent puncture attempts until a new catheter is successfully placed [14]. To maintain catheter patency, international standards on infusion therapy recommend the recurrent performance of PIVC flushing, usually with sodium chloride 0.9%, before drug administration (to assess device patency), between multiple drug administrations (to prevent the occurrence of incompatibilities between substances), and after drug delivery (to clean and maintain catheter functioning) [15,16,17]. Currently, this whole process is performed using two or more syringes, which increases the time and costs of intravenous therapeutics, as well as PIVC manipulation.

**Figure 1:**
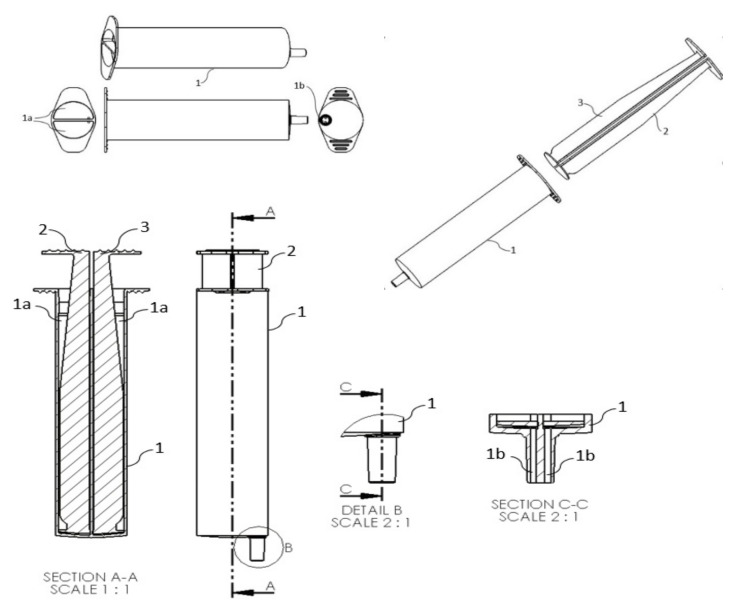
Double-chamber syringe (patent submission number 20192000899107 to Portuguese Authority in Industrial Property; international patent application number PCT/IB2020/056219 under the Patent Cooperation Treaty).

To address this challenge, a new medical device was developed to allow intravenous drug administration and the flushing procedure, as recommended in international standards of infusion nursing [15,16,17]. With input from the nurse end-users that prepare and administer intravenous drugs regularly, a new double-chamber syringe was developed, with a single 20 mL barrel divided by a vertical plate into two chambers (with two plungers, presented in two independent channels to prevent the mixture of solutions). The two 10 mL chambers display plungers with different colors to facilitate the identification of the flushing and the drug chambers. Visually, this double-chamber syringe is very similar to the syringes traditionally found on the market, enhancing healthcare professional’s sense of familiarity with the device and their ease of use. The two chambers have been developed with equal volumes of 10 mL each to accomplish the general requirements of intravenous therapeutics and the international standards for flushing procedures, which recommend a minimum flush volume equal to twice the internal volume of the entire vascular access device system (catheter, extension set and/or needless injection system) [15]. Given that each independent chamber can be used without restrictions, this double-chamber syringe allows healthcare professionals to perform a catheter flush before and after drug administration (Figure 1), which is considered good clinical practice in infusion therapy [15,17,18].

According to specific Portuguese legal requirements [19], implemented by the Portuguese National Authority of Medicines and Health Products (INFARMED) under European directives [20,21], this double-chamber syringe was classified as a class I device (sterile, with a measurement function). This new medical device has several advantages. Along with the reduced number of syringes used for intravenous therapeutics, this syringe will reduce the time required for the whole process of preparing and delivering intravenous therapeutics, but also the number of catheter manipulations that can lead to several related complications such as vascular trauma, infiltration, occlusion, or mechanical phlebitis.

Engineering/ergonomics of human factors play an important role in the medical devices’ development process [22,23], during the phases of design, prototyping and manufacturing [24]. Considering the current European directives, a medical device refers to any apparatus, software, material, or other similar or related item intended to be used in the diagnosis, prevention, monitoring, treatment or alleviation of a disease or injury [20,21]. The search for medical solutions that improve patient well-being and reduce healthcare costs has been responsible for the fast-growing industry in these contexts [24], with associated challenges concerning medical devices’ assessment. There are specific requirements for the Health Technological Assessment (HTA) of medical devices when comparing them with other health-related technologies [25,26,27,28]. 

Usability methods must be implemented to define and design new products based on end-users’ needs and functional requirements [24], but also to validate potential prototypes in simulated settings or real-life environments to create the final device [29]. Usability is defined by the International Organization for Standardization (ISO) as “the extent to which a system, product or service can be used by specified users to achieve specified goals with effectiveness, efficiency and satisfaction in a specified context of use” (ISO 9241-11: 2018) [30]. Usability evaluation is intended to provide information about the medical devices’ effectiveness and efficiency, as well as end-users’ satisfaction, as required by the legal authorities [21], being highly useful during product development in the determination of significant outcomes concerning the interaction between the end-users and a given product [31]. 

The main purpose of usability testing is to refine and validate a medical devices’ proposed design [32], through an early prototype, working model, production-equivalent device, or marketable device [33]. Usually, these are pre-clinical validation studies, whose main purpose is to determine whether a given medical device will meet its intended users’ needs and preferences through the performance of representative tasks as a means to provide evidence of the strengths and opportunities for device improvement [33]. Usability tests can be used to determine design priorities, evaluate learning effects, assess legibility of written instructions or icon clarity, as well as explore other design options [33]. Through these tests, developers may improve the usability of a specific device, maximizing users’ satisfaction and safety during its use [29,34], also reducing device recalls and the need for ad hoc modifications [35]. Hence, it is important to identify and recruit representative users, whose selection criteria should be carefully well-defined to seek a secure and reliable data set [36]. The conceptual foundation of usability testing resides on the Human-Centered Design (HCD) model [29,37] since it provides information about potential design concerns, an important aspect for validation in performance requirements such as quality, efficiency or safety in healthcare systems [22,38]. Usability assessment involves several qualitative and quantitative methods [25,35,39] that should be implemented along with the technological and industrial development of the MD [33,39,40]. 

This study aims to assess the usability of an innovative double-chamber syringe, by their nurse end users, through a mix-method of quantitative and qualitative data, considering the development stages (concept, semi-functional and functional prototype). Due to the lack of standardized and validated usability measures for the assessment of medical devices’ usability, we also attempted to develop and provide a first exploratory validation of a usability questionnaire.

## 2. Materials and Methods

### 2.1. Design and Procedures

In the last decade, the HCD has been used in medical devices’ development [23,29,33] to comply with international standards in this field [ISO 14155:2011; ISO 14971:2012] [41,42]. According to this design, after the definition of initial specifications for the double-chamber syringe, based on the international recommendations and information provided by their nurse end-users, the technological development of this new medical device was also supported by an iterative methodology based on a mix-method design of qualitative and quantitative studies on the devices’ usability. This iterative methodology was implemented along with the initial stages of product development. 

During the initial stages of concept and semi-functional prototype evaluation, focus groups with nurses were done to establish requirements and contexts of use, produce and assess design solutions, select the better design solution, and identify any modifications needed to improve the double-chamber syringe [18]. The participants also completed usability questionnaires focused on the design concept and a semi-functional prototype. Then, pre-clinical usability tests with the functional prototype were conducted through a two-arm parallel randomized controlled trial (RCT) in a simulated setting that enabled nurses to perform intravenous drug administration in a PIVC inserted on an upper arm simulator. The pre-clinical usability tests were performed in two phases: Phase (I): 10 nurses started the procedure using the double-chamber syringe and then the standard syringes, 10 nurses started the procedure using the standard syringes and then the double-chamber syringe; Phase (II): 10 nurses used the double-chamber syringe during the entire procedure; 10 used the standard syringes during the entire procedure. All the participants that used the double-chamber syringe during the usability tests were interviewed afterwards and completed a usability questionnaire.

### 2.2. Sample

According to the United States of America Food and Drug Administration (FDA), 15 participants per group or a minimum of 25 users should be enough to identify 90–97% of the usability problems [33]. Despite this, Borsci and colleagues [36] highlight that no definitive number of users equals reliable testing and consider that the number of users needed depends on the participants’ performance in identifying potential challenges/problems. Thus, concerning the usability tests with the double-chamber syringe, sample size was calculated according to the recommendations on ISO 62366-2 [43], which stipulates that a minimum sample of 10 users ensures the detection of potential challenges/problems and diminishes product returns. This is based on calculations determining the cumulative probability of detecting a usability problem: R = 1 − (1 − P) × *n*, where R = cumulative probability of detecting a usability problem, P = probability of a single test showing a usability problem, and *n* = number of participants. To comply with such standards, at least 10 nurses were involved in each stage of this study with the following inclusion criteria: have a bachelor degree (minimum academic title required); experience on intravenous drug administration; no previous contact with the newly developed double-chamber syringe (knowledge of the device or manipulation) and without any financial relationship with the device manufacturer and/or distributor. Thus, 16 nurses were involved during the concept stage, 22 nurses during the semi-functional prototype stage and 30 nurses during the testing of the final prototype (Table 1). To accomplish participants’ recruitment, the research team sent invitations to local tertiary hospitals in order to recruit nurses that meet the inclusion criteria previously presented.

### 2.3. Usability Questionnaire

Some usability questionnaires have been validated and developed for European Portuguese, such as the System Usability Scale (SUS) for products and users’ interfaces usability [44,45], as well as the Post-Study System Usability Questionnaire (PSSUQ) for system usability [46,47,48]. Moreover, a Usability Scale was developed based on the International Classification of Functioning, Disability and Health (ICF), the ICF-Usability Scale (ICF-US), specifically as part of a comprehensive framework for the design, development, and evaluation of ambient assisted living products and services for older adults [49,50]. However, in Portugal, there is a lack of standardized and validated usability measures specifically validated or developed for the assessment of medical devices’ usability in their development process. To address this gap, we attempted to develop and provide a first exploratory version of a usability questionnaire to be used in medical devices’ usability evaluation by their end-users in the several stages of product development. The usability questionnaire was developed through literature review and the revision of several assessment instruments for a selection of the initial pool of items for each dimension, namely: SUS [44,45]; PSSUQ [46,47,48]; Quebec User Evaluation of Satisfaction with Assistive Technology (QUEST 2.0) [51]; USE Questionnaire [52]; After Scenario Questionnaire (ASQ) [53,54]; Rating Scale Mental Effort (RSME) [55]. The Technology Acceptance Model (TAM) was used to establish a number of items concerning end-users’ technology acceptance and intention of use dimensions [56,57,58]. In the development of this questionnaire, the specificity of the medical devices regarding their efficacy, performance, and safety were considered as important features for a new medical device acquisition and use [59]. The expertise of academic, technological and industrial partners involved in medical devices’ development was also considered during the creation of an initial pool of items for the questionnaire to ensure its content validity. 

### 2.4. Ethics

This study was reviewed and approved (Ref. P608-8/2019) by the Ethics Committee of the Health Sciences Research Unit: Nursing (UICISA: E) of the Nursing School of Coimbra (ESEnfC). Eligible nurses received all the information about the study and provided written informed consent and signed a non-disclosure agreement (NDA). All legal aspects regarding privacy and confidentiality were ensured with ID alphanumeric codes for the participants, which were used in all data collection instruments. Study participants’ ID codes and data were anonymized for analysis and publications.

### 2.5. Data Analysis

Demographics (gender, age), educational (academic degree) and professional (clinical experience, work setting) data from the participants were entered and analyzed using the Statistical Package for the Social Sciences–version 22.0 (SPSS 22.0, SPSS Inc., Chicago, IL, USA). For quantitative data, means, standard deviations, frequencies, and percentages were used as descriptive statistics. Cronbach alpha, item-total, and item-domain correlations were calculated. Given that the four domains of the usability questionnaire had a different number of items, the global score of each domain was divided by the number of items that composed it, ensuring an equally balanced scale. For qualitative data, after the transcription of the individual interviews and focus groups, the content analysis technique was used [60] to identify end-users’ perceptions regarding the double-chamber syringe’s usability.

## 3. Results

### 3.1. Usability Questionnaire Development and Pilot Study

A usability questionnaire was developed with 42 items in a 7-point Likert scale (from 1—totally disagree to 7—totally agree), within four distinct domains (i) usefulness; (ii) ease of use; (iii) ease of learning; (iv) satisfaction and intention to use (Table 2, Table 3, Table 4 and Table 5).

The internal consistency of the 42-items usability questionnaire was deemed excellent (α = 0.976), and the item-total correlation values are also good (all above 0.30), which indicates a good internal consistency of the questionnaire. The higher correlation coefficients (>0.80) were observed in the *Satisfaction/intention to use* domain (8 items), one in the *Usefulness* and one in the *Ease of use* domains. In the *Ease of learning* domain the item-total correlations were between 0.335 (*p* < 0.05) and 0.740 (*p* < 0.01). Considering the specific values in each dimension, the item-domain correlations were also deemed good (higher than 0.50). Several items presented a correlation coefficient higher than 0.90, four in the *Satisfaction/intention to use* domain and one in the *Usefulness* domain. The only item that increased the questionnaire’s internal consistency if deleted was number 28 (“there is no need for written instructions to use it”), although this was considered negligible.

### 3.2. Double-Chamber Syringe Usability

The adjusted global scores (total and domains) of the usability questionnaire are presented in Table 6, considering the specific stages of product development (concept evaluation, semi-functional prototype evaluation, and functional prototype evaluation). Throughout the different stages, usability scores were consistently higher than the mean ponderation value of the 7-point Likert scale, generally indicating a good usability evaluation of the double-chamber syringe.

Qualitative data collected during the focus groups (in concept and semi-functional prototype stages) and individual interviews (in functional prototypes stage) was also positive in regards to the double-chamber syringe’s usability. Nurses emphasized the usefulness of this innovative device, highlighting the potential of the syringe “to assess the patency” and “to prevent drug interaction or contamination”. Furthermore, nurses stated that the syringe “reduces the amount of material wasted” and “reduces the number of catheter manipulations”, while “improving safety for patients and professionals” and “also lowers the risk of infection”. Regarding its ease of use, after manipulating the semi-functional and functional prototypes, nurses indicated the syringe body dimensions are “as close as possible to the syringes on the market”, emphasizing their simplicity in preparation and administration phases (“easier”, “reduce errors in the chambers charging”). Nurses also considered that a different color for each drug chamber facilitates the syringe’s use. 

However, during the functional prototype usability assessment, major concerns were also identified. Considering the total scores of the usability questionnaire, the mean values for the concept and semi-functional prototype stages were similar (M = 5.80 points), with a slightly lower value for the functional prototype (M = 5.28 points). The mean values of all usability domains are lower for the functional prototype when compared with the previous stages. This may be explained by major concerns highlighted by the participants when focused on the syringe’s ease of use, mainly in the drug preparation phase (“a bit more hand strength than usual is required”; “you feel slight pressure, making it harder to aspirate”; “having two plungers makes it harder to aspirate the solutions and remove aspirated air from the chamber”; “hand coordination is slightly harder with two plungers”). Despite the difficulties identified, most nurses recognized that “it is a matter of training and getting used to it”. In fact, regarding their ease of learning, the nurses confirmed that written instructions are valuable “to understand which chamber should be filled first” to avoid cross-chamber contamination.

During the different stages, the correlation coefficients between the usability scores and professional experience (measured in months) did not reach any statistical significance in all the three stages of product development (*p* > 0.05).

## 4. Discussion

The production of a clinically effective and safe medical device is not sufficient [39], and end-users involvement is important because it enables the early identification of their requirements, design needs, and recommendations [24]. In new devices development, usability assessment ensures that prototypes meet all the requirements of effectiveness, efficiency, safety and satisfaction [38,61] to move forward to clinical studies in real settings [62]. Thus, this study aimed to evaluate the usability of a new device (double-chamber syringe for infusion therapy through vascular access devices) considering the several stages of their development (concept, semi-functional prototype, and functional prototype). Nurses were considered the primary group of end-users that could provide significant inputs during the device’s development stages. 

Usability questionnaires play an important role in usability testing, along with other quantitative and qualitative methods [32,35,39]. Although a few usability questionnaires have been developed [49,50] and validated [45,48] for European Portuguese, there is no specific instrument for the evaluation of medical devices’ usability. Facing this, a new usability questionnaire was developed, and a pilot validation study was conducted. The involvement of the relevant stakeholders (academic, technological and industrial partners) along with a comprehensive review on existing scales and measures, ensured the content validity of the questionnaire. The item-total and item-domain correlation coefficients are positive and statistically significant, demonstrating the conceptual relevance of the items. Despite the importance of the quantitative data on usability, qualitative methods also enable the acquisition of more detailed and comprehensive data on the device being developed. Conceptual design solutions are important for product development, but the manipulation of a prototype, even semi-functional, enables the evaluation of significant aspects of the device’s usability features. Functional prototypes allow the end-users to perform a more rigorous evaluation of the medical device, by considering the relevant characteristics of its applications in real-life settings (e.g., hospital ward), which is not possible with semi-functional prototypes. In this study, the usability scores of the functional prototype were lower than in previous stages focused on the double-chamber syringe’s concept and semi-functional prototype. The quantitative data collected converges with the qualitative results gathered during the focus groups and interviews with nurses. Participants that use the functional prototype of the double-chamber syringe in a simulated setting identified significant challenges in its use. 

The incorporation of usability testing in medical devices’ development allows the early identification of end-users perceptions of a device’s effectiveness, ease of use, learning and training requirements [35]. This type of qualitative and quantitative data obtained during the product development cycle provides a common language for developers, end-users, and all stakeholders involved. Specifically, usability data will be used to ensure important outcomes for product development, as found in this study with a new medical device, or even highlight the need to redesign the device. A redesign can be defined as designing something again or in a different way, altering a product, but can also involve simple or subtle modifications of the product [59]. In this study, the quantitative and qualitative data collected provided a comprehensive overview of the double-chamber syringe from the nurses’ point of view, informing the research team of current challenges that must be addressed by the technological and industrial partners. 

Regarding the usability questionnaire, despite the satisfactory statistical results obtained, future validation studies are warranted with larger and heterogeneous samples, focused on the usability of several medical devices in different stages of product development, with different classifications and clinical applications. Given its current format and scoring system, future validation studies should implement different psychometric approaches to the questionnaire’s item validation, such as the Item Response Theory, since 3-point rating scales are considered more appropriate in the measurement of mental load and mental effort [63,64]. Moreover, future studies should be conducted to determine the usability cut-off-points for medical devices.

## 5. Conclusions

The development of innovative medical devices plays an important role in disease management and health promotion, providing healthcare professionals with efficient resources that can be used in the provision of safer care. Despite this, there are several risks for medical devices’ manufacturers, which can be associated with a lack of involvement of end-users within the medical devices’ development process. Usability testing is currently a legal requirement for medical devices development before placing it on the European Union market. In this context, quantitative data from usability questionnaires and qualitative information from focus groups/interviews provide a quick and simple usability testing during the development process of new medical devices, ensuring important information for all stakeholders involved in those development processes (designers, engineers, manufacturers).

## Figures and Tables

**Table 1:** Focus Groups: Users’ characterization.

	Concept Stage (*n* = 16)	Semi-Functional Prototype Stage (*n* = 22)	Functional Prototype Stage (*n* = 30)
Sex *n* (%)			
Male	5 (31.3%)	7 (31.8%)	8 (26.7%)
Female	11 (68.7%)	15 (68.2%)	22 (73.3%)
Age (years)	39.25 ± 10.096	37.86 ± 9.083	36.57 ± 8.012
M ± SD (Min.–Max.)	25–55	25–55	26–55
Education *n* (%)			
Bachelors’ degree	3 (18.7%)	3 (13.6%)	17 (56.7%)
Post-graduate/Specialty	4 (25.0%)	6 (27.3%)	4 (13.3%)
Master’s degree	9 (56.3%)	12 (54.5%)	7 (23.3%)
Ph.D.	-	1 (4.5%)	2 (6.7%)
Professional time (months)	195.56 ± 120.434	185.32 ± 106.512	163.87 ± 97.604
M ± SD (Min.–Max.)	25–55	36–372	24–384
Department *n* (%)			
Operating room	5 (31.3%)	6 (27.3%)	2 (6.7%)
General hospital	1 (6.2%)	1 (4.5%)	5 (16.7%)
Internal medicine	-	3 (13.6%)	3 (10.0%)
Research unit	3 (18.8%)	3 (13.6%)	2 (6.7%)
Cancer unit	1 (6.2%)	2 (9.1%)	2 (6.7%)
Gastroenterology	-	-	2 (6.7%)
Emergency Room	-	-	3 (10.0%)
Orthopaedics	2 (12.5%)	2 (9.1%)	1 (3.3%)
Rheumatology	-	-	2 (6.7%)
Intensive care unit	2 (12.5%)	2 (9.1%)	-
Physical medicine/Rehabilitation	-	-	2 (6.7%)
Pneumology	1 (6.2%)	1 (4.5%)	-
Haematology	-	-	1 (3.3%)
Pain consultation	-	1 (4.5%)	-
Psychiatric ward	-	-	1 (3.3%)
Burn unit	1 (6.2%)	1 (4.5%)	-
Urology	-	-	1 (3.3%)
Continued care unit	-	-	1 (3.3%)
Unemployed	-	-	1 (3.3%)
Type of healthcare institution *n* (%)			
Public institutions	13 (81.3%)	19 (86.4%)	25 (83.3%)
Private institutions	-	-	2 (6.7%)
Other (Teaching/Research)	3 (18.7%)	3 (13.6%)	3 (10.0%)
Time at the current professional unit (months)	135.25 ± 124.509	124.55 ± 110.403	185.32 ± 106.512
M ± SD (Min.–Max.)	6–372	6–372	1–384

M—Mean; SD—Standard Deviation; Min.—Minimum; Max.—Maximum.

**Table 2:** Usefulness items (The use of a double-chamber syringe for intravenous therapeutics).

Items	Item-Total Correlation	α (If Item Excluded)	Item-Domain Correlation
1. is useful for my work.	0.645 **	0.975	0.771 **
2. facilitates the performance of my tasks.	0.751 **	0.975	0.890 **
3. helps me to be more effective.	0.754 **	0.975	0.901 **
4. helps me to be more efficient.	0.781 **	0.975	0.897 **
5. achieves everything I would expect it to do.	0.763 **	0.975	0.803 **
6. allows me to complete my tasks.	0.728 **	0.975	0.767 **
7. allows me to complete my tasks easily.	0.763 **	0.975	0.814 **
8. allows me to complete my tasks quickly.	0.717 **	0.975	0.797 **
9. allows me to have better control over my tasks.	0.725 **	0.975	0.835 **
10. helps me to be more productive.	0.777 **	0.975	0.844 **
11. allows me to provide safer care.	0.661 **	0.975	0.751 **
12. answers my needs.	0.837 **	0.975	0.860 **

** *p* < 0.01.

**Table 3:** Ease of use items (The double-chamber syringe for intravenous therapeutics).

Items	Item-Total Correlation	α (If Item Excluded)	Item-Domain Correlation
13. is easy to use.	0.795 **	0.975	0.861 **
14. is simple to use.	0.815 **	0.975	0.870 **
15. is user-friendly.	0.721 **	0.975	0.802 **
16. requires few steps to accomplish my work.	0.670 **	0.975	0.815 **
17. is flexible to use according to my needs.	0.701 **	0.975	0.663 **
18. does not require physical effort to use it.	0.761 **	0.975	0.837 **
19. does not require mental effort to use it.	0.509 **	0.975	0.653 **
20. allows me to complete tasks in a logical sequence.	0.573 **	0.975	0.702 **
21. is not associated with significant possibilities of error in its use.	0.651 **	0.975	0.717 **
22. allows me to recover from mistakes quickly and easily.	0.651 **	0.975	0.642 **

** *p* < 0.01.

**Table 4:** Ease of learning items (Concerning the double-chamber syringe for intravenous therapeutics).

Items	Item-Total Correlation	α (If Item Excluded)	Item-Domain Correlation
23. I learned to use it quickly.	0.633 **	0.975	0.888 **
24. I learned to use it easily.	0.740 **	0.975	0.853 **
25. I easily remember how to use it.	0.611 **	0.975	0.867 **
26. I quickly became skilful with it.	0.651 **	0.975	0.863 **
27. it is not necessary too much previous knowledge to use it.	0.490 **	0.976	0.766 **
28. there is no need for written instructions to use it.	0.335 *	0.977	0.705 **

** *p* < 0.01; * *p* < 0.05.

**Table 5:** Satisfaction/Intention to use items (Concerning the possibility of using the double-chamber syringe for intravenous therapeutics in the future).

Items	Item-Total Correlation	α (If Item Excluded)	Item-Domain Correlation
29. I will be satisfied with it.	0.898 **	0.974	0.917 **
30. I would recommend it to colleagues.	0.884 **	0.974	0.914 **
31. it will allow the performance of my tasks.	0.875 **	0.974	0.902 **
32. it will be interesting for the performance of my tasks.	0.867 **	0.974	0.915 **
33. I feel I need to have it in my work.	0.608 **	0.974	0.656 **
34. it will be pleasant to use.	0.810 **	0.975	0.823 **
35. I will feel comfortable in using it.	0.851 **	0.975	0.861 **
36. I will feel confident in using it.	0.821 **	0.975	0.863 **
37. I will feel secure in using it.	0.788 **	0.975	0.835 **
38. the dimensions of the device are adjusted.	0.804 **	0.975	0.814 **
39. the weight of the device is adjusted.	0.479 **	0.976	0.577 **
40. the appearance of the device is adjusted.	0.531 **	0.976	0.560 **
41. I will like to use it frequently.	0.798 **	0.975	0.893 **
42. it will be easy to adjust it during the performance of my work.	0.725 **	0.975	0.802 **

** *p* < 0.01.

**Table 6:** Evaluation of the MD: Quantitative scores.

Usability Dimensions		Concept (*n* = 16)	*r*	Semi-Functional Prototype (*n* = 22)	*r*	Functional Prototype (*n* = 30)	*r*
Global score	M ± SD	5.80 ± 0.534	−0.174	5.80 ± 0.786	−0.250	5.28 ± 0.944	0.273
	Min.–Max.	4.81–6.76		3.93–7.00		3.19–7.00	
Usefulness	M ± SD	6.11 ± 0.593	0.062	5.95 ± 0.875	−0.283	5.36 ± 1.160	0.309
	Min.–Max.	4.75–7.00		3.58–7.00		2.00–7.00	
Ease of use	M ± SD	5.69 ± 0.597	0.267	5.72 ± 0.928	0.082	5.06 ± 1.013	0.183
	Min.–Max.	4.30–6.40		3.20–7.00		3.20–7.00	
Ease of learning	M ± SD	6.05 ± 0.767	−0.306	5.96 ± 0.916	0.111	5.53 ± 1.022	0.130
	Min.–Max.	4.33–7.00		4.00–7.00		2.83–7.00	
Satisfaction/Use intention	M ± SD	5.93 ± 0.860	−0.201	5.83 ± 0.826	−0.070	5.37 ± 1.082	0.274
	Min.–Max.	4.71–7.00		3.57–7.00		3.07–7.00	

M—Mean; SD—Standard Deviation; Min.—Minimum; Max.—Maximum; *r*—correlation coefficients with professional experience (months).
